# Splanchnic vein thrombosis following renal transplantation: a case report

**DOI:** 10.1186/1471-2369-14-161

**Published:** 2013-07-22

**Authors:** Erhan Tatar, Adam Uslu, Ahmet Aykas, Funda Tasli, Ozgur Oztekin, Gulsum Akgun Cagliyan

**Affiliations:** 1 Department of Nephrology and Transplantation Center, Izmir Education and Research Hospital, Izmir, Turkey; 2 Department of General Surgery and Transplantation Center, Izmir Education and Research Hospital, Izmir, Turkey; 3 Department of Pathology, Izmir Education and Research Hospital, Izmir, Turkey; 4 Department of Radiology, Izmir Education and Research Hospital, Izmir, Turkey; 5 Department of Hematology, Izmir Education and Research Hospital, Izmir, Turkey

**Keywords:** Thrombosis, Portosplenic vein thrombosis, JAK2 gene mutation, Myeloproliferative disorders, Renal transplantation

## Abstract

**Background:**

Recurrent episodes of venous thrombosis have been closely correlated with JAK2 V617F mutation. Upto date, JAK2 gene mutation has not been defined as a prothrombic risk factor in renal transplant recipients. Herein; we present a case of portosplenic vein thrombosis in a primary renal transplant recipient with JAK2 V617F mutation who had no history of prior venous thromboembolism or thrombophilia.

**Case presentation:**

A 59 year old female caucasian patient with primary kidney transplant admitted with vague abdominal pain at left upper quadrant. Abdominal doppler ultrasound and magnetic resonance imaging angiography demonstrated splanchnic vein thrombosis (SVT). The final diagnosis was SVT due to MPD (essential thrombocytosis, ET) with JAK2 V617F mutation. After 3 months of treatment with warfarin (≥5 mg/day, to keep target INR values of 1.9-2.5), control MRI angiography and doppler USG demonstrated partial (>%50) resolution of thrombosis with recanalization of hepatopedal venous flow. The patient is still on the same treatment protocol without any complication.

**Conclusion:**

JAK2 V617F mutation analysis should be a routine procedure in the diagnosis and treatment of kidney transplant patients with thrombosis in uncommon sites.

## Background

Kidney transplantation for the management of end-stage renal disease (ESRD) is still the best approach that provides near-normal renal function, excellent survival outcomes and improved quality of life with lowest healthcare costs [1]. This picture is occasionally overshadowed by posttransplant thromboembolic events which negatively influence graft and patient survival. A hypercoagulable state early after transplanation may cause deep venous thrombosis and induces devastating complications like pulmonary embolism and death [2-4].

The description of Janus Kinase 2 (JAK2) gene at the short arm of chromosome 9, brought new insight into thromboembolic events. JAK2 encodes a cytoplasmic tyrosine kinase of growth factors and cytokines like erythropoietin, thrombopoietin, granulocyte-macrophage colony-stimulating factor (GM-CSF) and interleukin-3 (IL-3) and functions as a co-factor for cytokine responses in vivo and in cytokine mediated transduction pathways leading to cell proliferation. A recent single point mutation substituting phenylalanine for valine (V617F) of JAK2 has resulted with dysregulation of kinase activity and consequent overactive hematopoesis [5,6]. Indeed, JAK2 V617F mutation frequently accompanied to chronic myeloproliferative disorders as polycythemia vera (PV) and essential thrombophilia (ET) in recent reports. Furthermore; patients with JAK2 V617F mutation have demonstrated recurrent episodes of venous thrombosis [5,7,8].

Splanchnic vein thrombosis (SVT) comprises extrahepatic portal vein, mesenteric vein and splenic vein thrombosis either alone or together and myeloproliferative diseases (MPD) are the most common etiologic factors [9]. Besides, JAK2 mutation has been accounted for the development of SVT, even in the absence of overt MPD [10]. Recent studies suggested JAK2 V617F mutation as a risk factor in the pathogenesis of SVT in general population [9,11] albeit JAK2 gene mutation as a prothrombic risk factor in renal transplant recipients is yet to be defined.

Herein; we present a case of portosplenic vein thrombosis in a primary renal transplant recipient with JAK2 V617F mutation.

## Case presentation

**Figure 1:**
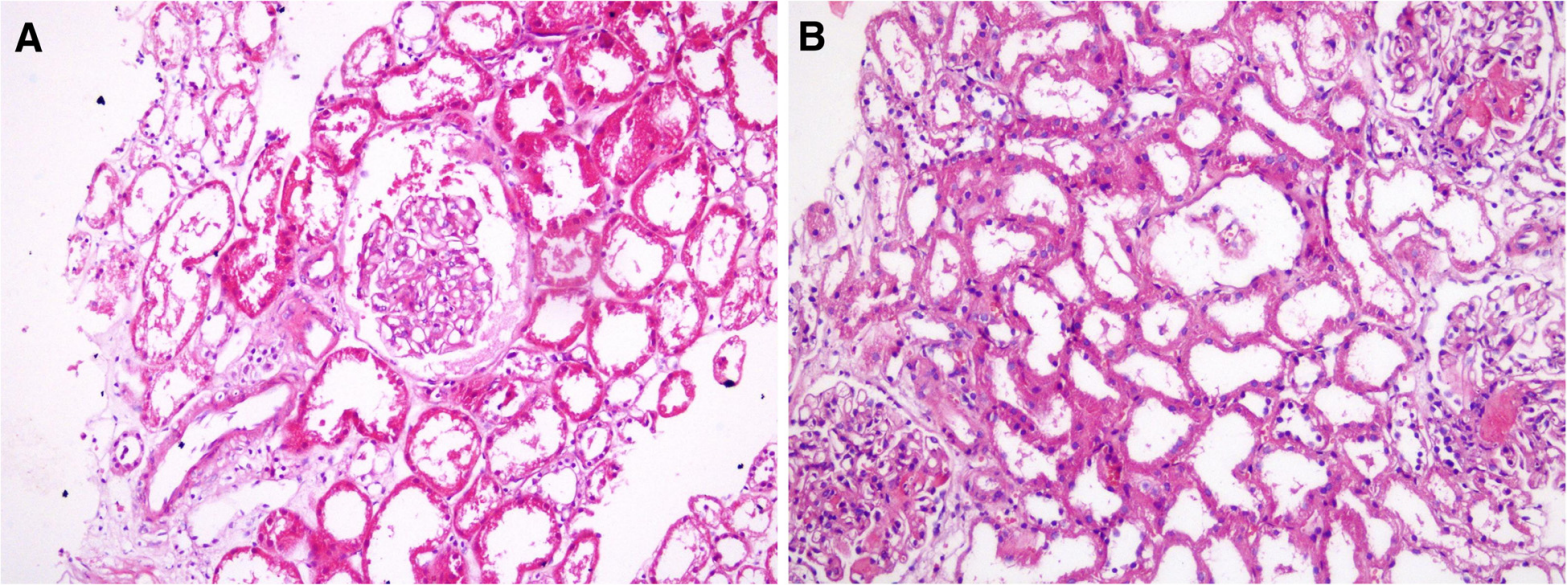
**A: Post-engraftment biopsy (moderate acute tubular injury with otherwise normal architecture) (HE, ×200). B**: Thrombotic microangiopathy, acute tubular necrosis (HE, ×200).

A 59 year old female ESRD patient with unknown etiology,underwent a preemptive living donor kidney transplant from her son on the 6th April 2011. The donor and recipient B and T-cell crossmatch was negative. She has been followed at the Nephrology Unit of our institution since August 2006. She received induction immunosuppression with rabbit anti-human thymocyte globulin (ATG-Fresenius, 3-5 mg/kg/day) with prednisolone and mycophenolic acid, until serum creatinine (SCr) values reduced to 2.0 mg/dL. She had excellent initial function with a SCr:0.94 mg/dL at the 3rd day. Post-engraftment biopsy specimen demonstrated normal morphology of glomeruli and vascular structures with moderate (25-50%) acute tubular necrosis (Figure 1A). The maintenance immunosuppression consisted of prednisolon 30 mg/day and mycophenolic acid 900 mg/day. A calcineurin inhibitor or a proliferation signal inhibitor (PSI) drug was not administered within the first week of transplantation due to profound immunosuppression of ATG, being reflected by very low CD3 + T-cell count. Post-transplant recovery was complicated by acute graft dysfunction at day 7. Kidney biopsy revealed de novo thrombotic microangiopathy (TMA) with characteristic fibrin thrombi within the glomerular capillaries and arterioles and endothelial swelling. There was no interstitial infiltration but features of recovering acute tubular necrosis. Immunoflourescence staining showed intracapillary fibrin thrombi in the glomeruli. C4d deposition in peritubular capillaries (PTCs) was not present (Figure 1B). Hematological parameters, peripheral blood smear and coagulation tests were normal with no noteworthy changes within the last few days.

The patient was treated with pulse methylprednisolone, Double-filtration plasmapheresis (every other day for three times) and administration of intravenous immunoglobulins (total dose:2 mg/kg). She had completely recovered in two weeks with good graft function (SCr:1.0 mg/dL and eGFR via MDRD4: 61 mL/min/1.73 m^2^.) and discharged from the hospital with a maintenance immunosuppression including prednisolone 10 mg/day, mycophenolic acid 900 mg/day and everolimus 0.5 mg/day. Her routine follow-up was normal until the outpatient visit at 20 months after transplantation. She admitted with vague abdominal pain principally located at left upper quadrant. Abdominal ultrasound with color-doppler imaging demonstrated splenomegaly with hyperechoic trombi almost completely occluding splenic vein and portal vein upto portal hilum. The spleen was 150 mm. in longitudinal axis and there were multiple areas of nonvascularized segments both in liver and spleen. Intraabdominal ascites and deep vein thrombosis in lower limbs or in other abdominal organs were not present. These findings were confirmed by abdominal magnetic resonance imaging angiography.

She had no history of prior venous thromboembolism or identifiable thrombophilia. Liver and kidney function tests were completely normal and quantitative HCV-RNA with CMV, HBV and BK virus DNA PCR testing were negative. The only abnormal finding was increased platelet count (500–650 × 10^3^/mm^3^) in two consecutive measurements preceding the SVT. There was no evidence of portal hypertension or esophageal varices in upper gastroduodenal endoscopy. The laboratory assessement, prothrombic risk factors and clotting parameters are shown in Table 1.

**Table 1:** Laboratory data, genetic screening and bone marrow analysis

	**Value**	**Reference range**
WBC count, ×10^3^/mm^3^	6.4	
Hgb level, g/L.	13.2	
Haematocrit, %	42.1	
Platelet count, ×10^3^/mm^3^	630	
**Peripheral blood smear**	**(%)**	
Neutrophils	67	
Lymphocytes	25	
Monocytes	6	
Eosinophils	1	
Basophils	1	
**Thrombophilia parameters**	**(%)**	(%)
Antithrombin III (AT III)	115.5	75-125
Protein C (PC)	98.4	70-140
Protein S (PS)	87.2	60-130
Activated PC resistance (APCR)	0.36	0.69-1.56
	(sec.)	
Lupus anticoagulant (LA)	45.1	31-34
Lupus anticoagulant confirmation	36.5	30-38
**Genetic screening**	**(pos./neg.)**	**Comment**
Factor V Leiden mutation	Positive	Heterozygot carrier
BCR-ABL Mutation	Negative	
prothrombin G20210A mutation	Negative	
JAK2 V617F mutation	Positive	
**Bone marrow biopsy**	**YES/NO**	
Increased cellularity	Yes	(60%)
Dyserythropoesis	No	
Dysgranulopoesis	No	
Megakaryocyte abnormality	Yes	Dysplastic changes
Clustering of megakaryocytes	Yes	Paratrabecular localization
Haemophagocytosis	No	
Fibrosis	No	

**Figure 2:**
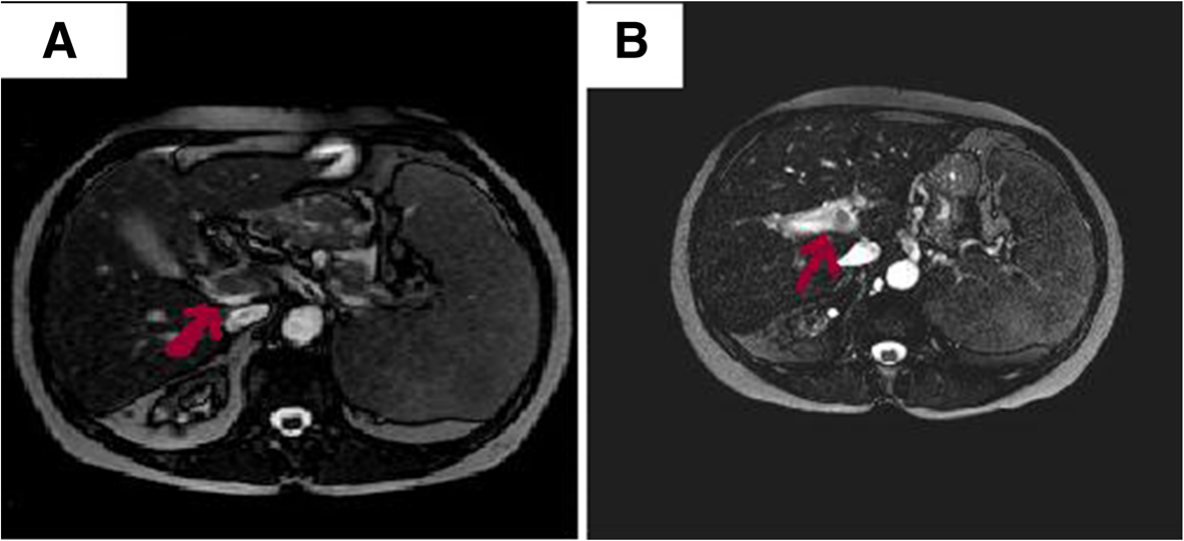
**A: MR imaging of portal vein thrombosis before anticoagulant treatment. B**: MRI of partial resolution of PV thrombus after warfarin treatment.

The patient was diagnosed as SVT due to MPD (essential thrombocytosis, ET) with JAK2 V617F mutation. She was initially treated with enoxaparin 60 mg/0.6 mL. b.i.d for three days. She received enoxaparin with warfarin 2.5 mg/day for the following two days and switched to warfarin (≥5 mg/day) afterwards to achieve target INR values of 1.9-2.5. After 3 months of treatment, control MRI angiography and Doppler USG demonstrated partial (>%50) resolution of thrombosis with recanalization of hepatopedal venous flow. (Figure 2A and B). There was no bleeding complication and the patient is still on the same treatment protocol.

## Discussion

The incidence of venous thromboembolism in renal transplant recipients, ranges between 0.6%-25% in the literature [2,4,12]. Many risk factors have been implicated in recurrent venous thrombosis or allograft thrombosis after renal transplantation. Among them; the presence of CMV infection and cyclosporine or rapamycine administration after transplantation are basically observations and the mechanism of thrombogenicity about these drugs and infection are not fully demonstrated in high-powered clinical trials [13-15]. In addition; pretransplant hypercoagulable states as vascular access thromboses, prior venous thromboembolism, essential thrombocytemia, ischemic heart disease on antiplatelet therapy, atrial fibrillation on vitamin K antagonist therapy, factor V Leiden and prothrombin G20210A mutation and the presence of antiphospholipid antibodies have all been defined as independant risk factors for renal allograft thrombosis but not for posttransplant SVT in patients with optimal graft function [13,16,17]. The impact of JAK2V617F mutation in the pathogenesis of splanchnic vein and peripheral venous thrombosis and thromboses in MPD has been disclosed in detail [5,7,8,18-20]. A very interesting finding in patients with thrombotic complications was the high prevalence of this mutation in female patients with SVT, indicating a gender-related susceptibility, while it was uncommon in patients with venous thrombosis at other locations or with arterial thrombosis [18,19]. There is strong clinical evidence that JAK2V617F mutation mediates overactivation of platelets in MPD [21,22]. In one retrospective study including 241 cases of SVT, the JAK2V617F mutation was identified in 34% of MPD patients with portal vein thrombosis (PVT). Here, the investigators suggestted JAK2V617F mutation analysis as an initial test which should replace bone marrow investigations for MPD in patients with SVT [20]. In another series of 99 adult patients with SVT but without an overt MPD, 15% presented JAK2V617F mutation [23]. This finding was reproduced and substantiated in another study group in which this somatic mutation was present in 100% of patients with SVT and overt MPD and 41% of patients with SVT and latent MPD. Interestingly none of the patients with latent MPD have progressed to overt MPD more than 7 years of follow-up [24]. The frequent co-existence of MPD, SVT and JAK2V617F mutation has directed us towards genetic analysis in our patient. The differential diagnosis of SVT late after transplantation in a recipient with normal graft function requires exclusion of the aforementioned risk factors or conditions that frequently contribute to venous thrombosis in common or uncommon locations. As shown in Table 1, this strategy is used in the present case. Factor V Leiden (G1691A) mutation was reported as a predisposing factor for thrombotic complications in renal transplant recipients. Genetic screening for protein C or S deficiency, antiphospholipid and anticardiolipin antibodies and G20210A prothrombin mutation were negative and JAK2V617F mutation was not studied in these patients [25].

The evaluation of etiology for SVT revealed JAK2V617F mutation accompanied by splenomegaly, thrombocytosis and bone marrow findings (megakaryocyte cellularity and clustering, presence of paratrabecular and dysplastic megakaryocytes without fibrosis) which indicates MPD, particularly essential thrombocytosis. The length of secondary prophylaxis after deep vein thrombosis (DVT) either by standard oral anticoagulation or by low molecular weight heparin is a matter of debate. In one study; 22 (46.8%) out of 47 patients with DVT experienced a recurrence after the withdrawal of antithrombotic treatment [2]. This high risk of recurrence in renal transplant patients should alert physicians for the administration of prolonged, probably life-long treatment, particularly to those with proven genetic susceptibility.

## Conclusion

To the best of our knowledge, this is the first case in the literature addressing the association between JAK2V617F mutation with MPD manifested by splanchnic vein thrombosis in a kidney transplant patient. This genetic study should be considered in kidney transplant patients with thrombosis in uncommon sites.

## Consent

Written informed consent was obtained from the patient for publication of this Case report and any accompanying images. A copy of the written consent is available for review by the Editor of this journal.

## Competing interests

The authors declare no competing interest.

## Authors’ contributions

ET and AU prepared the manuscript and searched literatures. AA assisted in the collection and interpretation of clinical and laboratory data. FT interpreted histopathological data. OO evaluated radiodiagnostic results. GAC evaluated hematologic analysis. All authors read and approved the final manuscript.

## Pre-publication history

The pre-publication history for this paper can be accessed here:

http://www.biomedcentral.com/1471-2369/14/161/prepub

## References

[B1] Jofre R Lopez-Gomez JM Moreno F Changes in quality of life after renal transplantation Am J Kidney Dis 1998 32 93 100 10.1053/ajkd.1998.v32.pm9669429 9669429

[B2] Poli D Zanazzi M Antonucci E Renal transplant recipients are at high risk for both symptomatic and asymptomatic deep vein thrombosis J Thromb Haemost 2006 4 988 992 10.1111/j.1538-7836.2006.01917.x 16689749

[B3] Kazory A Ducloux D Acquired hypercoagulable state in renal transplant recipients J Thromb Haemost 2004 91 646 10.1160/TH03-09-0568 15045124

[B4] Heidenreich S Junker R Wolters H Outcome of kidney transplantation in patients with inherited thrombophilia: data of a prospective study J Am Soc Nephrol 2003 14 234 239 10.1097/01.ASN.0000039567.22063.9D 12506156

[B5] Levine RL Pardanani A Tefferi A Gilliland DG Role of JAK2 in the pathogenesis and therapy of myeloproliferative disorders Nat Rev Cancer 2007 7 673 683 10.1038/nrc2210 17721432

[B6] James C Ugo V Le Couedic JP A unique clonal JAK2 mutation leading to constitutive signaling causes polycythemia vera Nature 2005 434 1144 1148 10.1038/nature03546 15793561

[B7] Baxter EJ Scott LM Campbell PJ Cancer Genome Project. Acquired mutation of the tyrosine kinase JAK2 in human myeloproliferative disorders Lancet 2005 365 1054 1061 15781101 10.1016/S0140-6736(05)71142-9

[B8] De Stefano V Fiorini A Rossi E Incidence of the JAK2 V617F mutation among patients with splanchnic or cerebral venous thrombosis and without overt chronic myeloproliferative disorders J Thromb Haemost 2007 5 708 714 10.1111/j.1538-7836.2007.02424.x 17263783

[B9] De Stefano V Martinelli I Splanchnic vein thrombosis: clinical presentation, risk factors and treatment Intern Emerg Med 2010 5 487 494 10.1007/s11739-010-0413-6 20532730

[B10] Riva N Donadini MP Dentali F Clinical approach to splanchnic vein thrombosis: risk factors and treatment Thromb Res 2012 130 Suppl 1 S1 S3 23026649 10.1016/j.thromres.2012.08.259

[B11] Donadini MP Dentali F Ageno W Splanchnic vein thrombosis: new risk factors and management Thromb Res 2012 129 Suppl 1 S93 S96 22682143 10.1016/S0049-3848(12)70025-7

[B12] Irish AB Green FR Environmental and genetic determinants of the hypercoagulable state and cardiovascular disease in renal transplant recipients Nephrol Dial Transplant 1997 12 167 173 10.1093/ndt/12.1.167 9027794

[B13] Irish A Hypercoagulability in renal transplant recipients. Identifying patients at risk of renal allograft thrombosis and evaluating strategies for prevention Am J Cardiovasc Drugs 2004 4 139 149 10.2165/00129784-200404030-00001 15134466

[B14] Lijfering WM de Vries AP Veeger NJ Possible contribution of cytomegalovirus infection to the high risk of (recurrent) venous thrombosis after renal transplantation Thromb Haemost 2008 99 127 132 18217144 10.1160/TH07-05-0340

[B15] Trotter JF Sirolimus in liver transplantation Transplant Proc 2003 35 193 200 10.1016/S0041-1345(03)00234-3 12742496

[B16] Ripert T Menard J Schoepen Y Preventing graft thrombosis after renal transplantation: a multicenter survey of clinical practice Transplant Proc 2009 41 4193 4196 10.1016/j.transproceed.2009.07.106 20005367

[B17] Murashima M Konkle BA Bloom RD A single-center experience of preemptive anticoagulation for patients with risk factors for allograft thrombosis in renal transplantation Clin Nephrol 2010 74 351 357 20979943 10.5414/cnp74351

[B18] Xavier SG Gadelha T Rezende SM JAK2V617F mutation in patients with thrombosis: to screen or not to screen? Int J Lab Hematol 2011 33 117 124 10.1111/j.1751-553X.2010.01275.x 21118380

[B19] Colaizzo D Tiscia GL Bafunno V Sex modulation of the occurrence of jak2 v617f mutation in patients with splanchnic venous thrombosis Thromb Res 2011 128 233 236 10.1016/j.thromres.2011.03.024 21497883

[B20] Kiladjian JJ Cervantes F Leebeek FW The impact of JAK2 and MPL mutations on diagnosis and prognosis of splanchnic vein thrombosis: a report on 241 cases Blood 2008 111 4922 4929 10.1182/blood-2007-11-125328 18250227

[B21] Austin SK Lambert JR The JAK2 V617F mutation and thrombosis Br J Haematol 2008 143 307 320 10.1111/j.1365-2141.2008.07258.x 19004076

[B22] Vannucchi AM JAK2 mutation and thrombosis in the myeloproliferative neoplasms Curr Hematol Malig Rep 2010 5 22 28 10.1007/s11899-009-0038-x 20425393

[B23] Xavier SG Gadelha T Pimenta G JAK2V617F mutation in patients with splanchnic vein thrombosis Dig Dis Sci 2010 55 1770 1777 10.1007/s10620-009-0933-y 19690956

[B24] Orr DW Patel RK Lea NC The prevalence of the activating JAK2 tyrosine kinase mutation in chronic porto-splenomesenteric venous thrombosis Aliment Pharmacol Ther 2010 31 1330 1336 10.1111/j.1365-2036.2010.04299.x 20331577

[B25] Wüthrich RP Cicvara-Muzar S Booy C Maly FE Heterozygosity for the factor V Leiden (G1691A) mutation predisposes renal transplant recipients to thrombotic complications and graft loss Transplantation 2001 72 549 550 10.1097/00007890-200108150-00037 11502996

